# Genetic and immunological insights into COVID-19 with acute myocardial infarction: integrated analysis of mendelian randomization, transcriptomics, and clinical samples

**DOI:** 10.3389/fimmu.2023.1286087

**Published:** 2023-11-06

**Authors:** Zequn Zheng, Yueran Zhou, Yongfei Song, Pengxiang Ying, Xuerui Tan

**Affiliations:** ^1^ Department of Cardiology, First Affiliated Hospital of Shantou University Medical College, Shantou, Guangdong, China; ^2^ Clinical Research Center, First Affiliated Hospital of Shantou University Medical College, Shantou, Guangdong, China; ^3^ Ningbo Institute for Medicine &Biomedical Engineering Combined Innovation, Ningbo, Zhejiang, China; ^4^ Centre for Precision Health, Edith Cowan University, Perth, WA, Australia

**Keywords:** COVID-19, acute myocardial infarction, causal relationship, TLR4, ABCA1, immune dysregulation

## Abstract

**Background:**

Globally, most deaths result from cardiovascular diseases, particularly ischemic heart disease. COVID-19 affects the heart, worsening existing heart conditions and causing myocardial injury. The mechanistic link between COVID-19 and acute myocardial infarction (AMI) is still being investigated to elucidate the underlying molecular perspectives.

**Methods:**

Genetic risk assessment was conducted using two-sample Mendelian randomization (TSMR) to determine the causality between COVID-19 and AMI. Weighted gene co-expression network analysis (WGCNA) and machine learning were used to discover and validate shared hub genes for the two diseases using bulk RNA sequencing (RNA-seq) datasets. Additionally, gene set enrichment analysis (GSEA) and single-cell RNA-seq (scRNA-seq) analyses were performed to characterize immune cell infiltration, communication, and immune correlation of the hub genes. To validate the findings, the expression patterns of hub genes were confirmed in clinical blood samples collected from COVID-19 patients with AMI.

**Results:**

TSMR did not find evidence supporting a causal association between COVID-19 or severe COVID-19 and AMI. In the bulk RNA-seq discovery cohorts for both COVID-19 and AMI, WGCNA’s intersection analysis and machine learning identified TLR4 and ABCA1 as significant hub genes, demonstrating high diagnostic and predictive value in the RNA-seq validation cohort. Single-gene GSEA and single-sample GSEA (ssGSEA) revealed immune and inflammatory roles for TLR4 and ABCA1, linked to various immune cell infiltrations. Furthermore, scRNA-seq analysis unveiled significant immune dysregulation in COVID-19 patients, characterized by altered immune cell proportions, phenotypic shifts, enhanced cell-cell communication, and elevated TLR4 and ABCA1 in CD16 monocytes. Lastly, the increased expression of TLR4, but not ABCA1, was validated in clinical blood samples from COVID-19 patients with AMI.

**Conclusion:**

No genetic causal link between COVID-19 and AMI and dysregulated TLR4 and ABCA1 may be responsible for the development of immune and inflammatory responses in COVID-19 patients with AMI.

## Background

Cardiovascular disease stands as the foremost cause of global mortality, with a substantial portion of deaths attributed to ischemic heart disease. The emergence of COVID-19, stemming from the SARS-CoV-2 virus, has introduced new complexities in the management of cardiovascular conditions (1). While COVID-19 primarily impacts the respiratory system, it also poses potential cardiovascular challenges, including myocarditis, heart failure, stress cardiomyopathy, acute myocardial infarction (AMI), and arteriovenous thrombosis (2, 3). Reports indicate varying prevalence rates of acute myocardial injury linked to COVID-19, spanning from 5% to 38%, with a concurrent rise in mortality (4). Notably, AMI, a form of heart attack that occurs when there is a sudden and severe reduction in blood flow to a part of the heart muscle, has been frequently observed among COVID-19 patients (5, 6). Among over 3000 COVID-19 inpatients, AMI emerged as the most prevalent atherothrombotic complication, manifesting in 8.9% of cases (7). Although a meta-analysis did not find a statistically significant difference in AMI admission rates during the pandemic (8), several cohort studies, including a comprehensive investigation in Sweden, have indicated that COVID-19 independently poses a notable risk for AMI (9, 10).

The precise underlying mechanisms of COVID-19-induced AMI remain uncertain. A hypothesis suggests that the excessive inflammatory immune response and cytokine storm triggered by SARS-CoV-2 might contribute to the development of systemic multisystemic complications, including AMI, resulting in an unfavorable prognosis for COVID-19 patients (11). Further investigation is needed into the communication between viral antigens and the release of cytokines and proinflammatory factors. Moreover, genome-wide association studies (GWAS) on COVID-19 have progressively revealed genomic loci associated with disease susceptibility and severity that are suggestive of underlying biological processes involving inflammatory signaling, immune metabolism, and blood coagulation (12, 13). Yet, the understanding of these genetic markers has not fully clarified the complex biological interactions between COVID-19 and its associated conditions. Whether these genetic factors play a role in the development of AMI remains uncertain.

In this study, we employed Mendelian randomization (MR) to deduce the potential causality between COVID-19 and AMI. This approach helps mitigate biases from observational studies and guards against the influence of reverse causality (14). Additionally, we utilized transcriptomics data including bulk RNA sequencing (RNA-seq) and single-cell RNA-seq (scRNA-seq) to identify plausible central molecules regulating the connection between COVID-19 and AMI. For validation, we collected clinical blood samples from COVID-19 patients with AMI. Our intention through these analyses is to offer genetic and immunological insights into COVID-19 with AMI.

## Materials and methods

### GWAS data sources

In the latest round (round 7; release date: April 8, 2022) of the GWAS on COVID-19 susceptibility and severity, as published by the COVID-19 Host Genetics Initiative (https://www.covid19hg.org), we obtained exposure-related data. The dataset encompassed a total of 2,597,856 individuals for COVID-19 susceptibility and 1,086,211 individuals for severe COVID-19 outcomes. The outcome GWAS data were sourced from Coronary ARtery DIsease Genome wide Replication and Meta-analysis (CARDIoGRAM) plus The Coronary Artery Disease (C4D) Genetics (CARDIoGRAMplusC4D) consortium (http://www.cardiogramplusc4d.org) and previously published related studies (15–17). The CARDIoGRAMplusC4D database incorporates the GWAS meta-analysis of coronary artery disease (CAD) conducted by Nikpey et al., which encompassed 184,305 CAD and 171,875 AMI cases (15), followed by a subsequent meta-analysis performed by Aragam et al. with an expanded cohort comprising 1,165,690 participants (16). In addition, the recently published GWAS by Hartiala et al. on 831,000 myocardial infarction subjects was included in our study (17). All the datasets included in our study pertained to European populations or populations predominantly of European descent. For clarity, we refer to COVID-19 and severe COVID-19 exposures as “Covid” and “Sevcovid,” respectively, while outcomes are designated by the names of the researchers: Aragam, Hartiala, and Nikpey. Ethical approval was unnecessary since our study involved reanalyzing publicly available and previously published data.

### MR analysis

We extracted single nucleotide polymorphisms (SNPs) as MR instrumental variables (IVs) in the GWAS of Covid and Sevcovid according to genome-wide significance (*P* < 5 × 10^-8^) and independence (LD r^2^ = 0.001 and kb = 10000). These IVs were chosen based on their strong correlation with the exposure but not with the outcome while being independent of potential confounders. Robust IVs correlation was confirmed with F-statistics exceeding 10. To satisfy the independence and exclusion restriction assumption of MR, SNPs associated with age, smoking, alcohol consumption, body mass index, blood glucose, lipid levels, and other confounders potentially affecting AMI were screened using the PhenoScanner online tool to not be considered as IVs. The TwoSampleMR package was utilized for two-sample MR (TSMR) and sensitivity analysis. Heterogeneity among the IVs was assessed with Cochran’s Q-statistic, and potential horizontal pleiotropy was examined using the Egger intercept test (*P* < 0.05). All analyses were conducted using R software (version 4.2.2).

### Bioinformatics data and clinical blood sample sources and processing

Independent RNA-seq datasets were accessed from the Gene Expression Omnibus (GEO) database. The bulk RNA-seq dataset was selected based on the presence of cases and controls, with each group containing at least 10 samples. The selected datasets for discovery cohorts were GSE66360 for AMI and GSE179850 for COVID-19, comprising 50 controls and 49 AMI patients, and 16 controls and 31 COVID-19 patients, respectively. Additionally, 17 AMI and 7 control samples from GSE60993 were merged with GSE97320 to create a pooled expression profile (GSEmer). GSEmer with 20 AMI cases and 10 control samples and GSE179627, which consisted of 48 COVID-19 patients and 22 controls, were used as validation cohorts. Batch normalization was applied to eliminate batch effects resulting from different annotation platforms. For the scRNA-seq dataset, an integrated dataset was formed by selecting two patient samples (GSM4557331 and GSM4557332) and two control samples (GSM4557337 and GSM4557338) from GSE150728 to check the immune response in the periphery to severe COVID-19. In addition, clinical peripheral blood samples were collected from 10 COVID-19 patients with AMI and 10 healthy individuals with the approval of the Ethics Committee of Ningbo University Affiliated Li Huili Hospital. Peripheral blood mononuclear cells (PBMCs) were harvested using Ficoll-Paque PREMIUM (#17544203, Cytiva, USA), and total RNA was isolated using the TransZol Up kit (#ET111-01-V2, TransGen, Beijing, China) and amplification was detected by reverse transcription-quantitative polymerase chain reaction (RT-qPCR).

### RT-qPCR

Reverse transcription was performed on 1 μg of RNA template from each sample using the HiScript III All-in-one RT SuperMix Perfect for qPCR kit (R333-01, Vazyme, Nanjing, China) as per the instruction manual. The cDNA that resulted was employed as a template for real-time PCR analysis. The ChamQ Universal SYBR qPCR Master Mix (Q711-02, Vazyme, Nanjing, China) and gene-specific primers were used for real-time PCR. An Applied Biosystems 7500 real-time PCR machine was used for the amplification. An initial denaturation phase of 2 minutes at 95°C was followed by 40 cycles of denaturation at 95°C for 10 seconds and annealing/extension at 65°C for 1 minute. Relative quantification was achieved by the 2^-ΔCq^ method, with β-actin as the housekeeping gene. The following primer sequences were used: TLR4: AGACCTGTCCCTGAACCCTAT (forward primer) and CGATGGACTTCTAAACCAGCCA (reverse primer), ABCA1: ACCCACCCTATGAACAACATGA (forward primer) and GAGTCGGGTAACGGAAACAGG (reverse primer), ACTB: CATGTACGTTGCTATCCAGGC (forward primer) and CTCCTTAATGTCACGCACGAT (reverse primer).

### Analysis of differential expression

Gene expression information was normalized and differentially expressed genes (DEGs) were obtained with the “LIMMA” package (version 4.2.1). DEGs were determined as |LogFC| > 0.5 for GSE66360 and |LogFC| > 1 for GSE179850. The R package “ggVolcano” was used for volcano mapping.

### Weighted gene co-expression network analysis (WGCNA) and determination of shared genes

WGCNA was conducted with the R package “WGCNA” (version 1.71). The analysis involved determining a suitable soft threshold (power) to transform the correlation matrix into an adjacency matrix for the construction of a co-expression network. The generation of a topological overlap matrix (TOM) with specific parameter values (minModuleSize = 30 and mergeCutHeight = 0.2) was undertaken to quantify similarity. Hierarchical clustering dendrograms were utilized to visualize genes based on TOM dissimilarity. Correlations between module eigengenes (MEs) and interested traits were assessed to identify modules associated with the disease. Gene significance (GS) and module membership (MM) correlation were employed to identify relevant modules. Shared genes related to AMI and COVID-19 were determined by extracting and intersecting the genes from modules that positively correlated with the MEs of clinical features. The R package “UpSetR” was utilized to visualize specific shared genes.

### Functional and pathway enrichment analysis

Gene Ontology (GO) and KEGG enrichment analyses were conducted on the shared genes identified in the WGCNA analysis through the online platform Sangerbox (http://www.sangerbox.com/tool). A significance threshold of *P* < 0.05 and FDR < 0.20 was applied.

### Prioritization and validation of common key genes

A protein-protein interaction (PPI) network was built with the “STRING” database, and hub genes were identified by Cytoscape’s plugin cytoHubba (version 3.9.1). Four algorithms (DMNC, MCC, MNC, and Degree) were used to predict the top 5 hub genes. Machine learning algorithms, Least Absolute Shrinkage and Selection Operator (LASSO) and Support Vector Machine Recursive Feature Elimination (SVM-RFE), were employed to further identify the most significant hub genes. The LASSO regression model was constructed using the “glmnet” package, with optimal λ values determined based on lambda.min. This allowed for the selection of feature genes with non-zero coefficients. On the other hand, the “caret” package, known for its feature selection capabilities, including the SVM-RFE algorithm, was employed. The “rfe” function within this package facilitated the creation of RFE models utilizing the “svmRadial” method to generate feature genes. The resulting genes were intersected to find common genes.

### Construction of the diagnostic and predictive model

Gene expression levels of common hub genes were extracted from the validation cohort. Statistical analysis was performed using the “ggplot2” package to illustrate expression differences between groups. The “pROC” package determined the optimal expression cut-off value, generating receiver operating characteristic (ROC) curves for the area under the curve (AUC) calculation. A combined diagnostic ROC curve was constructed via a multivariable logistic regression model. Micro- and macro-averaged ROC curves for multivariable prediction were produced using the “multi_roc” function. Furthermore, a prognostic nomogram model was established using the “lrm” and “regplot” packages to assess predicted disease risk based on gene expression. Nomogram performance was evaluated using Harrell’s concordance index (C-index). Decision curve analysis (DCA) utilized the “decision_curve” function from the “rmda” package, encompassing simple models with individual genes and complex models incorporating gene pairs. The analysis was designed as a “case-control” study, with the threshold range set from 0 to 1.

### Single-gene gene set enrichment analysis (GSEA) and single-sample GSEA (ssGSEA)

GSEA allows the function of molecules to be explored in gene sets with the same expression pattern. To perform single-gene GSEA in a list of 428 genes from the turquoise module of COVID-19, we first used the “cor” function in R to calculate batch correlations between the selected hub genes and the other genes. The resulting list of genes was then ordered by correlation coefficients and used for single-gene GSEA analysis. This analysis was achieved with the “GSEA” function in the package “clusterProfiler”, annotated as “c2.all.v7.0.entrez.gmt”. The resulting pathways that contained the hub gene were then visualized. Scores from ssGSEA were calculated using the “gsva” function in the package “GSVA” (version 1.46.0), with the method specification set as “ssgsea” (18).

### scRNA-seq analysis pipelines

The R package “Seurat” (version 4.3.0) was utilized for scRNA-seq data preprocessing and analysis. Data scaling, transformation, and quality checking were all part of the preparation stages. Cells with fewer than 200 or more than 6,000 unique genes, as well as those exceeding 20% mitochondrial content, were excluded. The four samples were merged into an integrated dataset, and the “Harmony” function was applied to remove batch effects. Variable genes were identified using the “SCTransform” function, and the first 20 principal components (PCs) derived from linear principal component analysis of these variable genes were utilized for nonlinear (UMAP) dimensionality reduction. Cell identity was determined by identifying DEGs for each cluster through the “FindAllMarkers” function, and manual verification was performed based on CellMarker2.0 (19) and PanglaoDB (20). The “FetchData” function was employed to extract gene expression values for statistical analysis and visualization in GraphPad Prism (version 9.2).

For cell-cell communication analysis within the PBMCs dataset, the “celltalker” package (version 0.0.7.9000) and “cellchat” package (21) (version 1.6.1) were utilized. Ligand-receptor interactions were identified in the disease or healthy control dataset based on cell types. Ligand-receptor interactions specific to disease or healthy control datasets were identified based on cell types. Interactions involving at least 100 cells, ligand expression between 1000 and 20000 counts, and interactions with FDR < 0.05 were considered significant. The three interactions with the highest interaction ratio for each cell type were selected for visualization in a circular plot, where ligands were denoted in blue and receptors in red. In the “celltalker” package, the “computeCommunProb” function was used for inferring interaction strength, and the “computeCommunProbPathway” function provided insights into communication at the signaling pathway level. The “aggregateNet” function enabled the calculation of the aggregated cell-cell communication network, and the “netAnalysis_computeCentrality” function facilitated the determination of network centrality scores.

## Results

### TSMR and sensitivity analysis

**Figure 1:**
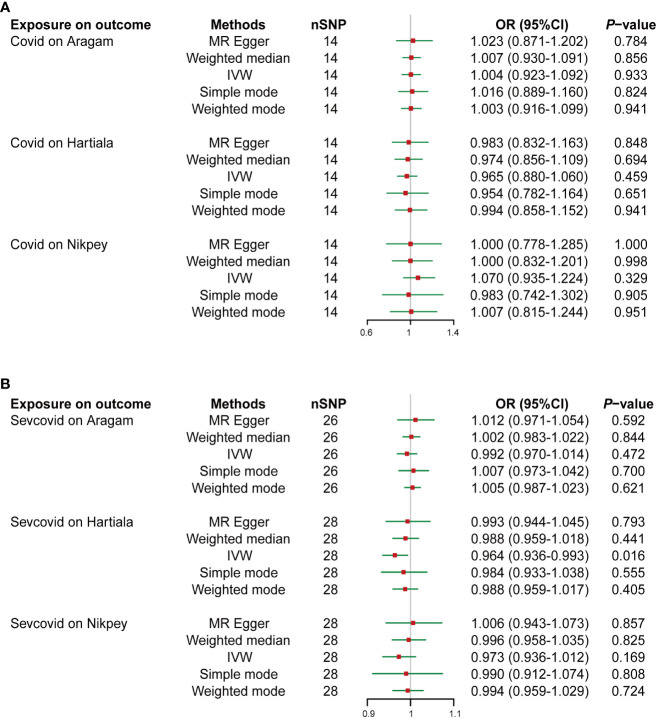
Two-sample Mendelian randomization (TSMR) analyses of COVID-19 and the risk of acute myocardial infarction (AMI). Summary statistics of genome-wide association studies in the COVID-19 (Covid) **(A)** or severe COVID-19 (Sevcovid) **(B)** cohorts were used for exposure and summary statistics of genome-wide association studies (GWAS) in the coronary artery disease (CAD) or AMI cohorts (Aragam, Hartiala, and Nikpey) were used for outcome analyses. The risk of exposure versus outcome was presented as odds ratio (OR). SNP, single nucleotide polymorphism; IVW, Inverse variance weighted; 95%CI, 95% confidence interval.

TSMR was employed to ascertain if COVID-19 serves as a heritable causal risk factor for AMI. Following the exclusion of rs554833 due to its strong association with the outcome, a total of 14 appropriate IVs were analyzed for Covid on three AMI GWAS cohorts (Details of the IVs are available in **Table S1**). In the case of Sevcovid, strongly outcome-associated rs550057 was excluded, and an additional two SNPs (rs289705 and sr17279437) incompatible with Aragam’s GWAS were also removed. Consequently, a final selection of suitable IVs was made for the Aragam, Hartiala, and Nikpey cohort, encompassing 26, 28, and 28 IVs, respectively (Details of the IVs are available in **Table S2**). Inverse variance weighted (IVW) results indicated that the odds ratio (OR) and 95% confidence intervals (95% CIs) for Covid across the three outcomes GWAS were 1.004 (0.923-1.092) (*P* = 0.933), 0.965 (0.880-1.060) (*P* = 0.459), and 1.070 (0.935-1.224) (*P* = 0.329) (**Figures 1A**, **S1A**). Similarly, when conducting the IVW analysis with Sevcovid as the exposure variable, the results suggested three ORs and their respective 95% CI: 0.992 (0.970-1.014) (*P* = 0.472), 0.964 (0.936-0.993) (*P* = 0.016), and 0.973 (0.936-1.012) (*P* = 0.169) (**Figures 1B**, **S1B**). Sensitivity analysis including heterogeneity and pleiotropy assessment further confirmed the stability of the results (**Table 1**). Collectively, these findings suggest that there is no genetically inferred causal association between COVID-19 and AMI.

**Table 1:** Sensitivity analysis results of Mendelian randomization for COVID-19 on acute myocardial infarction (AMI).

Exposure	Outcome	Heterogeneity			Pleiotropy		
		Method	Q-value	*P*-value	Method	Value	*P*-value
Covid	Aragam	MR Egger	27.386	0.007	Egger intercept	-0.001	0.783
		IVW	27.567	0.010			
	Hartiala	MR Egger	9.185	0.687	Egger intercept	-0.001	0.800
		IVW	9.252	0.754			
	Nikpey	MR Egger	9.156	0.690	Egger intercept	0.004	0.545
		IVW	9.545	0.731			
Sevcovid	Aragam	MR Egger	67.934	4.47E-06	Egger intercept	-0.004	0.277
		IVW	71.439	2.35E-06			
	Hartiala	MR Egger	50.190	0.003	Egger intercept	-0.006	0.174
		IVW	53.954	0.002			
	Nikpey	MR Egger	46.157	0.009	Egger intercept	-0.007	0.213
		IVW	49.045	0.006			

### Identification of DEGs in COVID-19 and AMI

**Figure 2:**
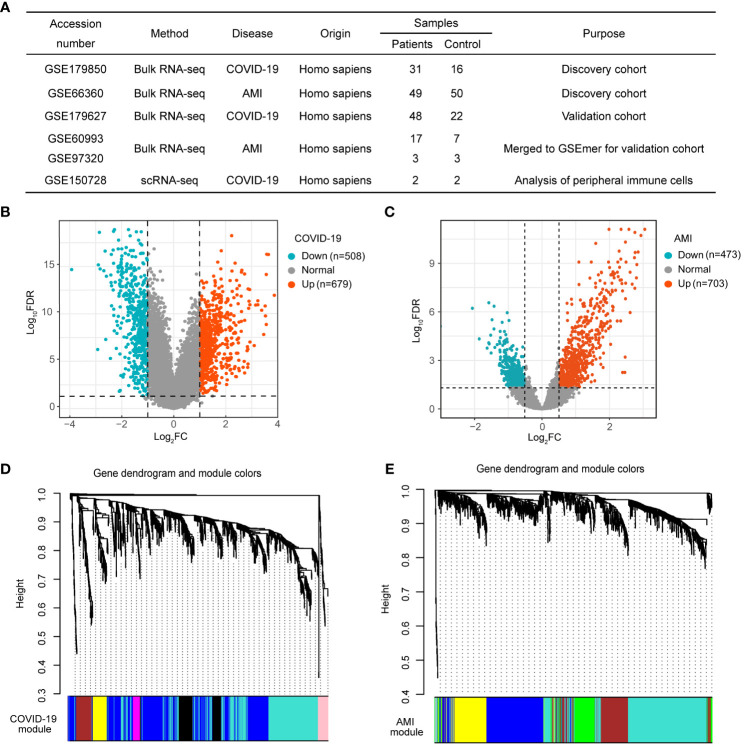
Weighted gene co-expression network analysis (WGCNA) of differentially expressed genes (DEGs) in COVID-19 and AMI. **(A)** Details of the GEO datasets involved in this study. **(B)** Volcano plot of DEGs in COVID-19 patients. **(C)** Volcano plot of DEGs in AMI patients. **(D)** Hierarchical clustering tree representing module-identification in COVID-19 co-expression patterns. **(E)** Hierarchical clustering tree representing module identification in AMI co-expression patterns.

The GEO database was used to retrieve the datasets used in this investigation, including the discovery cohort, validation cohort, and scRNA-seq dataset (**Figure 2A**). The datasets were processed using pre-defined criteria, and DEGs were found and visualized using volcano plots in the discovery cohort. COVID-19 was determined to have 1187 DEGs, with 679 up-regulated and 508 down-regulated genes (**Figure 2B**). In the case of AMI, 1176 DEGs were found, with 703 being up-regulated and 473 being down-regulated (**Figure 2C**).

### Characterization of COVID-19 and AMI-associated gene modules

**Figure 3:**
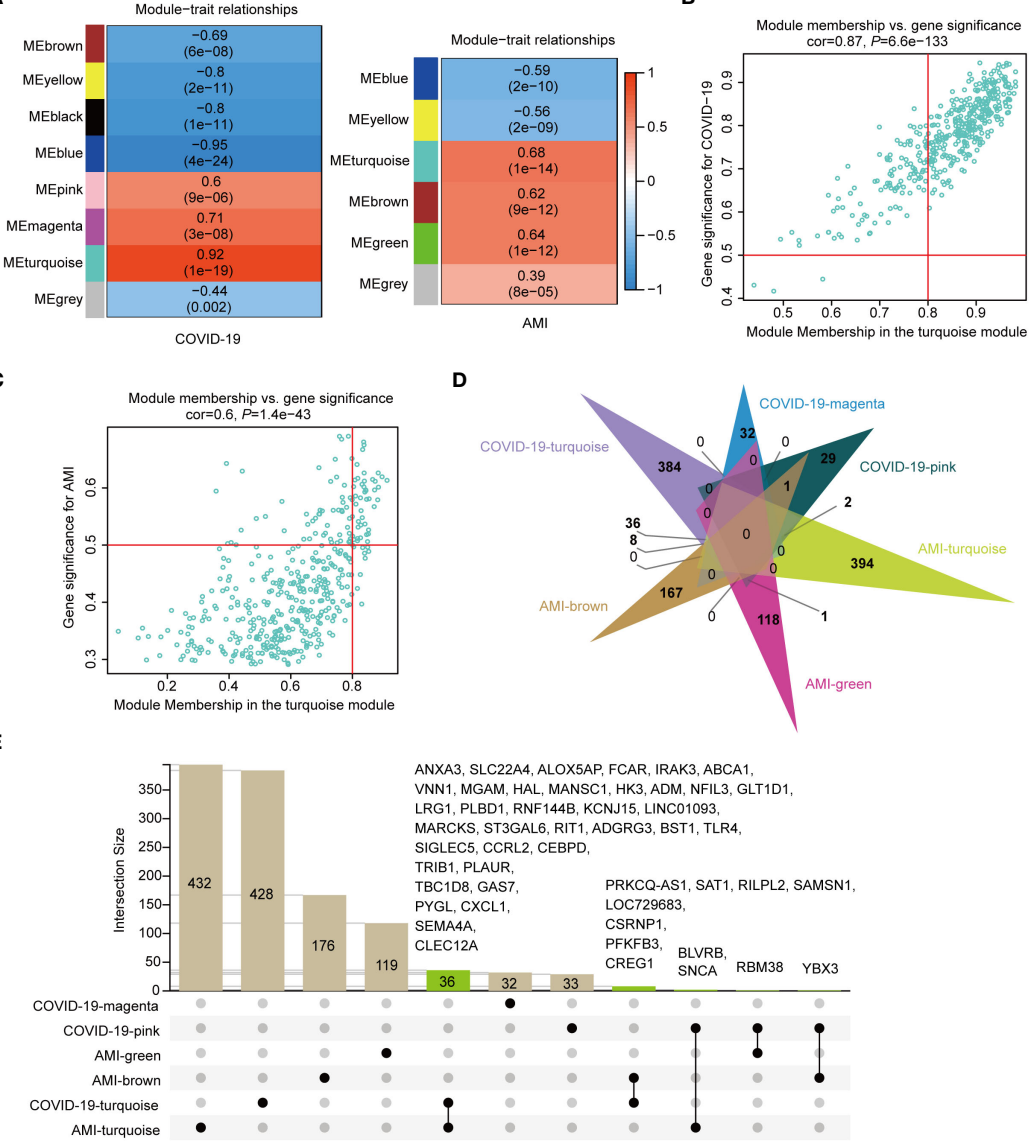
Shared genes of COVID-19 and AMI patients identified in disease-associated modules. **(A)** Correlation of module eigengenes (MEs) in COVID-19 and AMI patients. **(B)** Correlation between gene significance (GS) and MEturquoise membership in COVID-19 patients. **(C)** Correlation between GS and MEturquoise membership in AMI patients. **(D)** Identification of the intersection genes in each module positively associated with COVID-19 and AMI. **(E)** Specific presentation of the intersecting genes by UpSetR. Each shared genetic symbol between the modules of the disease is presented.

WGCNA was used to study the link between DEGs and clinical traits and also to discover co-expressed gene modules. We selected optimal βs of 11 and 5 for the construction of COVID-19 and AMI scale-free networks, respectively. Gene clustering on TOM-based dissimilarity for co-expression networks yielded 8 modules for COVID-19 (**Figure 2D**) and 6 modules for AMI (**Figure 2E**). After the exclusion of the grey module, module correlation analysis showed that three modules (turquoise, magenta, and pink) were strongly positively correlated with COVID-19, with the turquoise module (428 genes) showing the greatest significance (**Figure 3A**). Similarly, three modules (turquoise, brown, and green) were positively correlated with AMI, with the turquoise module (432 genes) being the most correlated (**Figure 3A**). Genes included in the turquoise module also correlated significantly with the gene significance (**Figures 3B, C**). These findings imply that the gene set in the turquoise module may play a critical biological role in both COVID-19 and AMI.

### Identification of genes shared by disease-associated modules

**Figure 4:**
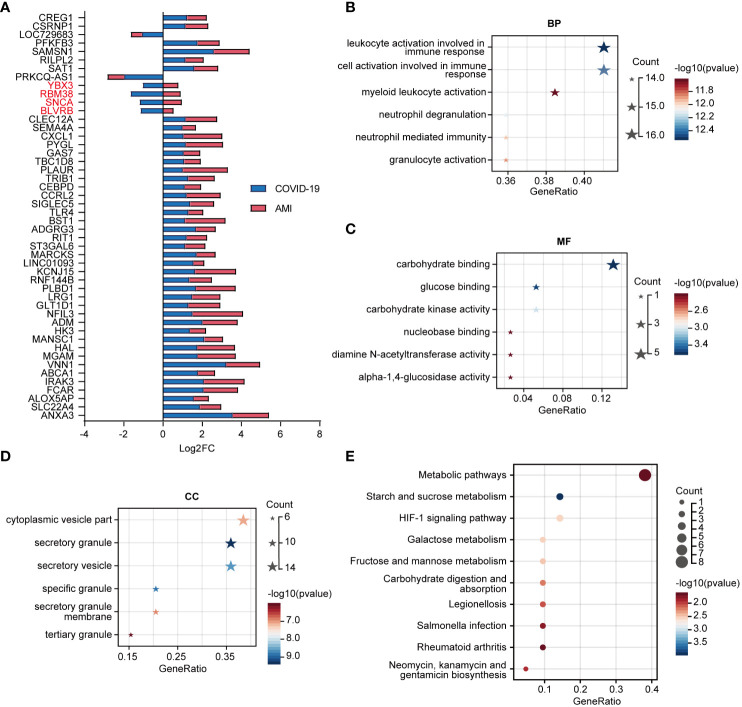
Fold change in expression of shared genes for COVID-19 and AMI and their functional and pathway enrichment analysis. **(A)** Shared gene expression fold changes (Log2FC) (disease *vs.* control). Four genes with opposite expression trends are marked in red. **(B)** Enriched Gene Ontology (GO) biological process (BP) of shared genes. **(C)** Enriched GO molecular function (MF) of shared genes. **(D)** Enriched GO cellular component (CC) of shared genes. **(E)** KEGG pathway enrichment analysis of shared genes.

To investigate disease-shared genes, we took the intersections in six modules positively associated with AMI and COVID-19 (**Figure 3D**). A total of 48 intersecting genes were identified, with two turquoise modules sharing 36 genes (**Figure 3E**). Subsequently, the fold change values of the 48 genes in their respective expression matrix were extracted (**Figure 4A**). By excluding 4 genes, YBX3, RBM38, ANCA, and BLVRB, with opposite expression trends, we found that when compared to control samples, practically all of these genes showed persistently high expression in AMI and COVID-19 (**Figure 4A**). The 44 shared genes were subject to subsequent analysis.

### GO function and KEGG pathway annotation of shared genes

We used GO function and KEGG signaling pathway enrichment analyses to discover the biological activities of the 44 genes shared by AMI and COVID-19 patients. The top 6 significantly enriched GO terms, including biological process (BP) (**Figure 4B**), molecular function (MF) (**Figure 4C**), and cellular component (CC) (**Figure 4D**) are presented. BP enrichment analysis showed that these genes were linked to immune cell activation pathways (**Figure 4B**), while MF, which provided the highest significant enrichment pathway, was linked to glucose metabolism (**Figure 4C**). The top three significant signaling pathways identified by KEGG analysis included the metabolic pathways and the HIF-1 signaling pathway (**Figure 4E**).

### Identification of the most significant hub genes among the shared genes

**Figure 5:**
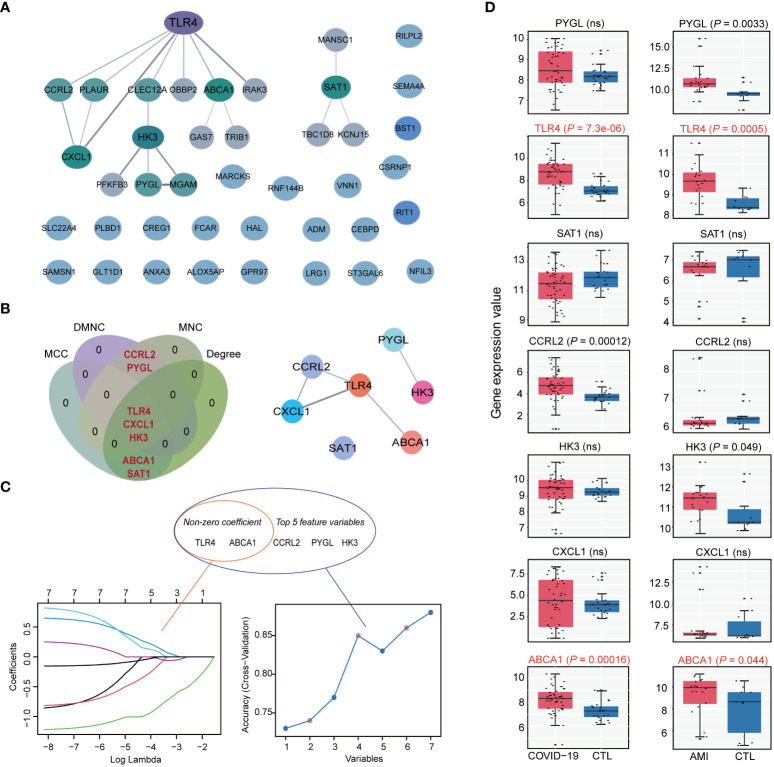
Identification of hub genes in shared disease genes. **(A)** Protein-protein interaction (PPI) network of disease-shared genes. **(B)** Venn diagram of intersecting hub genes identified by the 4 algorithms of cytoHubba from the PPI network and the merged networks of hub genes. **(C)** Machine learning algorithm based on Least Absolute Shrinkage and Selection Operator (LASSO) (left) and Support Vector Machine Recursive Feature Elimination (SVM-RFE) (right) to select the most significant feature genes from intersecting hub gene. LASSO identified 2 genes with non-zero coefficients, whereas SVM-RFE identified and selected the top 5 feature genes. **(D)** Expression values of intersecting hub genes in the validation cohorts of COVID-19 (COVID-19, n=48; CTL, n=42) and AMI (AMI, n=20; CTL, n=10). Statistically significant differences in both COVID-19 and AMI (*P* < 0.05) are marked as red. ns: non-significant.

To find hub genes, a PPI network was built utilizing the shared genes. The network consisted of 41 nodes and 19 edges (**Figure 5A**). Four algorithms (MCC, Degree, DMNC, and MNC) were employed to determine the top 5 hub genes in the network, yielding 7 overlapping genes: PYGL, MGAM, TLR4, CXCL1, HK3, ABCA1, and SAT1 (**Figure 5B**). Machine learning algorithms such as LASSO and SVM-RFE were used to screen feature variables from the set of 7 overlapping hub genes to identify the most significant hub genes. LASSO identified two genes with non-zero coefficients, TLR4 and ABCA1, while SVM-RFE also indicated TLR4 and ABCA1 as the most relevant genes among the top 5 selected variables (**Figure 5C**). We investigated these genes’ expression patterns further in the validation cohort and discovered that two genes, TLR4 and ABCA1, showed statistical differences in both the COVID-19 and AMI cohorts (**Figure 5D**). Therefore, TLR4 and ABCA1 were determined to be the most significantly correlated genes in common with COVID-19 and AMI.

### Diagnostic and predictive value of TLR4 and ABCA1

**Figure 6:**
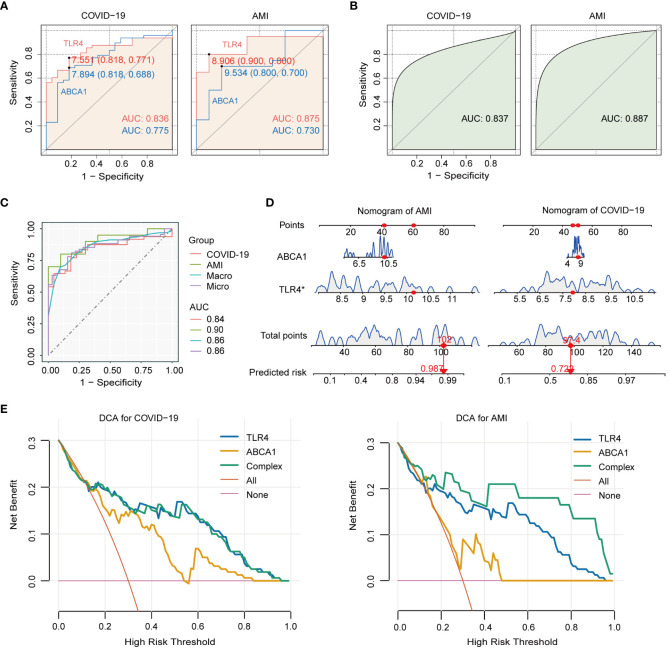
Diagnostic value of TLR4 and ABCA1 genes in COVID-19 with AMI and their single-gene GSEA analysis. **(A)** ROC analysis of TLR4 and ABCA1 genes in COVID-19 and AMI. **(B)** ROC analysis of TLR4 combined with ABCA1 in COVID-19 and AMI. **(C)** Macro- and micro-averaged ROC analysis of TLR4 combined with ABCA1 in COVID-19 with concurrent AMI. **(D)** Nomogram predicting risk for AMI and COVID-19 by ABCA1 and TLR4. **(E)** The decision curve analysis (DCA) model derives the net benefit. The complex model (Complex) was constructed by incorporating TLR4 and ABCA1 as joint predictive factors.

The discriminative ability of TLR4 and ABCA1 between illness and control groups was assessed using ROC curves. The results showed that TLR4 and ABCA1 had moderate to good diagnostic values in COVID-19 and AMI, with TLR4 performing better than ABCA1 (AUC in COVID-19: TLR4, 0.836; ABCA1, 0.775. AUC in AMI: TLR4, 0.875; ABCA1, 0.730.) (**Figure 6A**). Logistic regression-based multivariable diagnoses yielded AUCs of 0.837 and 0.887 for the two genes in COVID-19 and AMI, respectively (**Figure 6B**). Furthermore, micro- and macro-averaged ROC curves were used to evaluate the diagnostic utility of COVID-19 with AMI, indicating a stable performance in the validation cohort, with a macro/micro mean AUC of 0.86 (**Figure 6C**). Nomogram models indicated the value of the two genes in predicting disease risk, with C-index for AMI and COVID-19 were 0.905 and 0.837, respectively (**Figure 6D**). Additionally, DCA was implemented to assess the clinical utility of various models in predicting disease outcomes. The net benefit curves plotted at different thresholds revealed clear separation from the extreme curves, indicating the clinical relevance of the models. The complex model combining TLR4 and ABCA1 consistently outperformed the four simple models, exhibiting higher net benefit within the threshold range of 0 to 1 (**Figure 6E**).

### Immunological correlation of TLR4 and ABCA1

**Figure 7:**
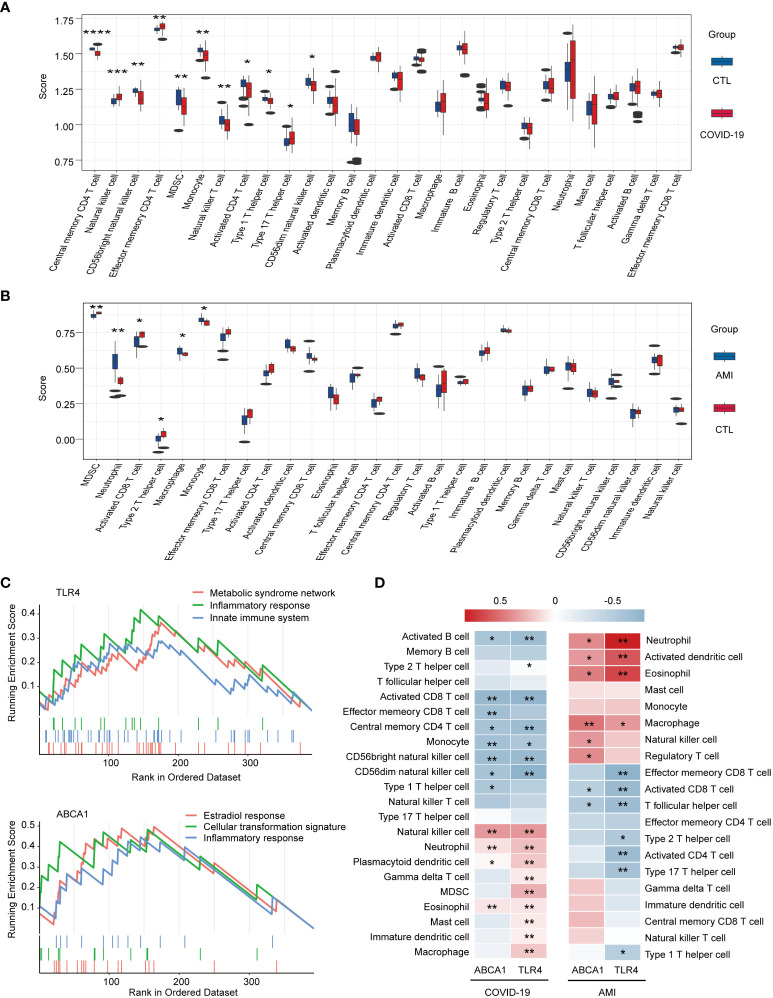
Immunocorrelation and immune cell infiltration analysis using single-gene GSEA and ssGSEA methods. **(A)** Box plot of immune scores for COVID-19 and healthy controls. **(B)** Box plot of immune scores for AMI and healthy controls. **(C)** Single-gene GSEA analysis of TLR4 and ABCA1. Signaling pathways involving TLR4 or ABCA1 are shown. **(D)** Heat map of the correlation between ABCA1 and TLR4 and immune cell infiltration. * *P* < 0.05, ** *P <*0.01, *** *P* < 0.001, **** *P* < 0.0001.

To further explore the relevance of TLR4 and ABCA1 to immunity, immune cell infiltration was measured through the ssGSEA score. Compared to healthy controls, the disease ssGSEA score showed that COVID-19 was most significantly associated with memory CD4 T cells (*P* < 0.0001) (**Figure 7A**), whereas AMI was most significantly associated with myeloid-derived suppressor cells (MDSC) (*P* < 0.01) (**Figure 7B**). Importantly, both COVID-19 and AMI were significantly associated with monocyte (*P* < 0.01 and *P* < 0.05) (**Figures 7A, B**). Furthermore, the results of the single-gene GSEA suggest that similar to GO enrichment analysis, TLR4 and ABCA1 were primarily associated with the activation of metabolic, immune, and inflammatory responses (**Figure 7C**). The heat map of gene-immune cell relationship revealed a positive link between ABCA1 and TLR4 in COVID-19 and AMI, particularly TLR4 with a range of immune cells including NK cells, neutrophils, eosinophils, dendritic cells, and macrophages (**Figure 7D**). These findings suggest that TLR4 and ABCA1 may play crucial roles in controlling the immunological response to COVID-19 and AMI, presumably by activating metabolic, immune, and inflammatory pathways and by modulating the infiltration of specific immune cell types.

### Immunological cell profiling and expression patterns of TLR4 and ABCA1 in scRNA-seq data

**Figure 8:**
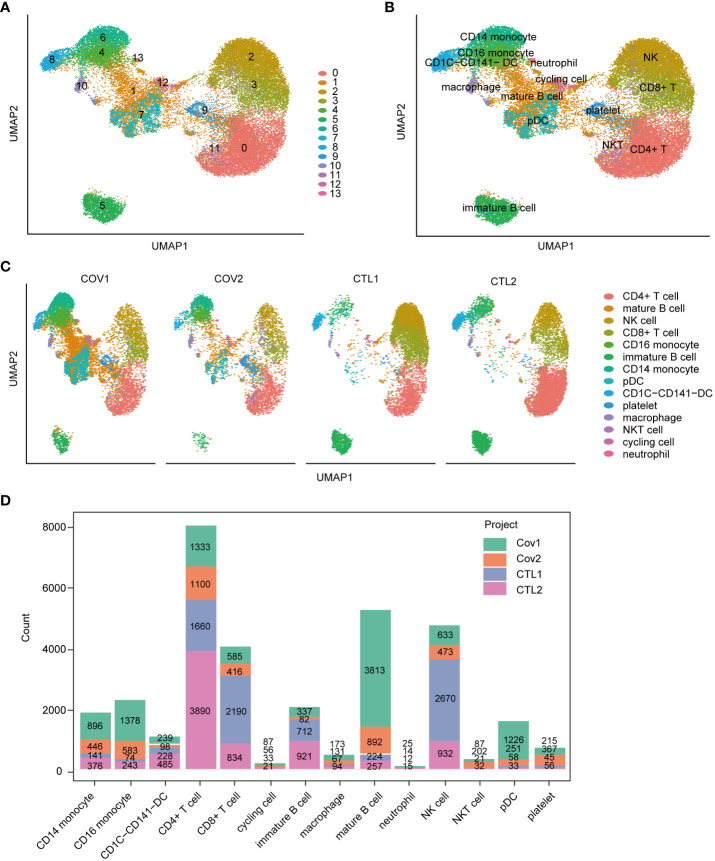
Immunological cell profiling of scRNA-seq data from peripheral blood samples of severe COVID-19 patients. **(A)** UMAP dimensionality reduction embedding for the integrated dataset of scRNA-seq data from all profiled samples (n = 32,462 cells) colored by inferred cluster identity. **(B)** UMAP embedding of the integrated dataset colored by orthogonally generated clusters labeled by manual cell type annotation. **(C)** UMAP grouped by donor of origin (COV1: COVID-19 sample #1; COV2: COVID-19 sample #2; CTL1: healthy control sample #1; CTL2: healthy control sample #2. **(D)** Bar chart representing the count of various cell types across different samples. pDC, plasmacytoid dendritic cell.

To investigate the expression patterns of ABCA1 and TLR4 in immune cells, we analyzed scRNA-seq data from PBMCs of COVID-19 patients and healthy controls. Four samples from disease and healthy controls were merged into a single integrated Seurat object containing 32462 cells and 36921 genes. We identified 14 distinct cell clusters with specific cellular identities using dimensionality reduction and graph-based clustering (**Figures 8A, B**). Grouped dimensionality reduction revealed significant phenotypic differences between COVID-19 patients and controls, most notably in monocytes, T cells, and NK cells (**Figure 8C**). COVID-19 patients showed a reduction in T cells and NK cells, along with an increase in monocytes and dendritic cells (DCs) (**Figures 8C, D**). Moreover, B lymphocytes in COVID-19 patients exhibited a shift toward mature phenotypes (**Figure 8D**), consistent with previous reports (22).

**Figure 9:**
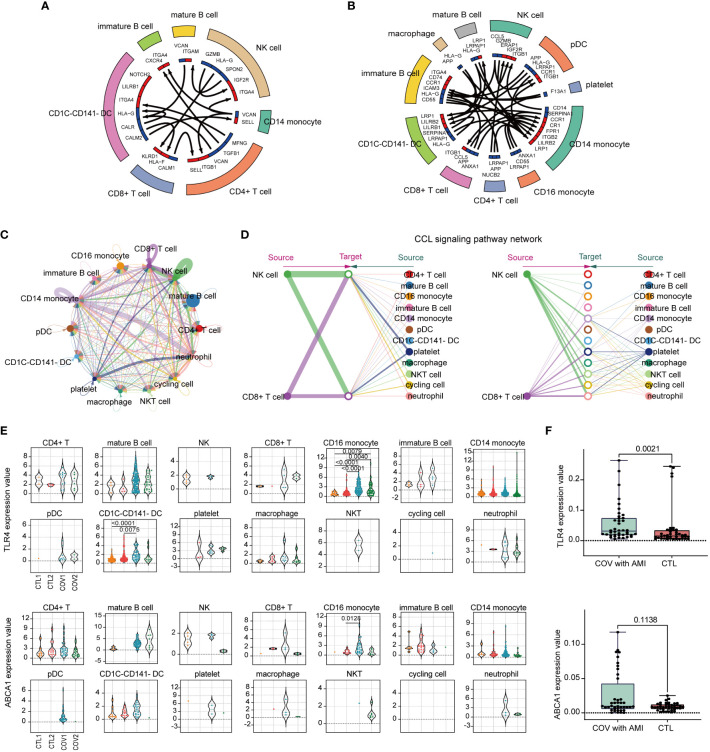
Analysis of immune cell-cell communication and expression patterns of TLR4 and ABCA1. **(A)** A circular plot of cell-cell communication patterns in scRNA-seq samples from healthy controls. **(B)** A circular plot of cell-cell communication patterns in scRNA-seq samples from severe COVID-19 patients. **(C)** An aggregated cell-cell communication network shows the number of interactions or total interaction strength between any two cell groups. **(D)** The signaling network of CCL (chemokine ligand) pathways and their communication patterns among different cell populations. **(E)** Violin plots colored by the donor of TLR4 and ABCA1 expression values for each cell type. **(F)** Identification of ABCA1 and TLR4 gene expression in peripheral blood from clinical patients with COVID-19 complicated with AMI by RT-qPCR, and healthy individuals served as controls. Ten samples each from disease and control were analyzed and each sample was repeatedly measured four times (Disease, n=40; CTL, n=40).

Cell-cell communication analysis indicated that compared with healthy controls (**Figure 9A**), severe COVID-19 cases (**Figure 9B**) had increased intercellular signaling and enhanced communication between CD14 monocytes and CD16 monocytes. The integrated cell-cell communication network analysis revealed that CD14 monocytes and NK cells exhibited the highest degree of communication weight, while platelets enhanced communication with other cell types in COVID-19 (**Figure 9C**). The chemokine ligand (CCL) signaling pathway was identified as a central contributor to intercellular communication between different cell populations (**Figure 9D**). Additionally, TLR4 and ABCA1 showed significant upregulation specifically in CD16+ monocytes of COVID-19 patients (**Figure 9E**), suggesting their potential involvement in the immune response during COVID-19.

### Identification of high expression of TLR4 in clinical blood samples from patients with COVID-19 complicating AMI

To examine the clinical significance of our findings, PBMCs were collected from COVID-19 patients with concurrent AMI and from healthy individuals. Gene expression levels were measured in these distinct groups. Consistent with our analysis of single-cell data, the results from PBMC analysis corroborated our findings. Particularly, TLR4 exhibited a significant upregulation in the PBMC samples from the patients (**Figure 9F**), emphasizing the crucial involvement of TLR4 in the disease’s pathogenesis.

## Discussion

**Figure 10:**
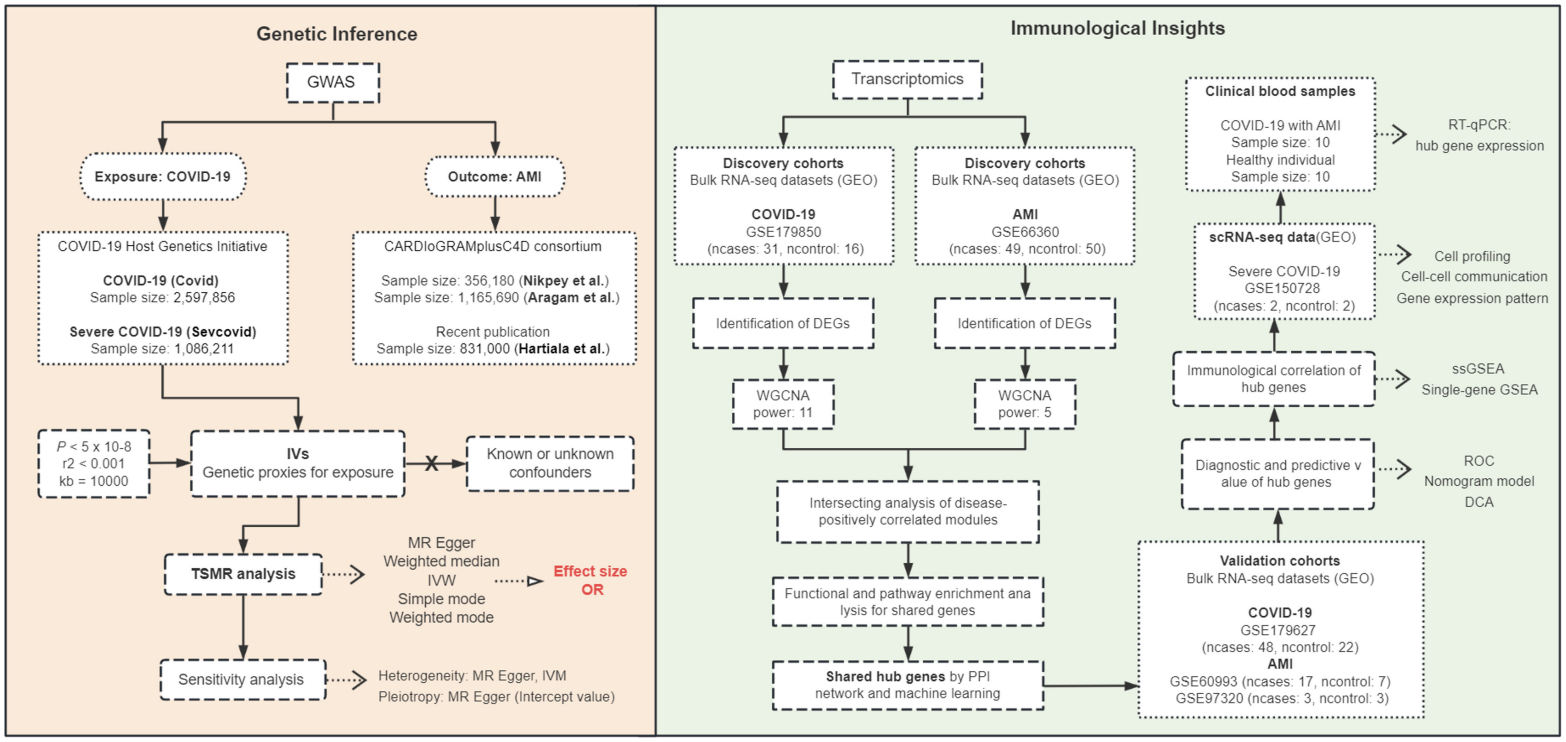
Workflow diagram of the study. Two-sample Mendelian randomization (TSMR) was used to infer causality between COVID-19 exposure and AMI outcome, and transcriptomics of bulk RNA sequencing (RNA-seq) and single-cell RNA-seq (scRNA-seq) and clinical blood samples from COVID-19 patients with AMI were analyzed to investigate the immunological mechanisms of the disease. GWAS, genome-wide association studies; IVs, instrumental variables; IVW, Inverse variance weighted; OR, odds ratio; GEO, Gene Expression Omnibus database; DEGs, differentially expressed genes; WGCNA, weighted gene co-expression network analysis; power, soft threshold; PPI, protein-protein interaction; RT-qPCR, reverse transcription-quantitative polymerase chain reaction; ssGSEA, single-sample gene set enrichment analysis; ROC, receiver operating characteristic; DCA, decision curve analysis.

The co-occurrence of COVID-19 and AMI may involve a cascade of responses triggered by SARS-CoV-2 infection causing systemic inflammation, immune cell hyperactivation, and a cytokine storm, which contribute significantly to the morbidity and mortality (2, 23). Within this intricate interplay, the comprehensive comprehension of the precise correlation between COVID-19 and AMI presents a formidable scholarly pursuit. Therefore, we employed TSMR for genetic inference to investigate whether COVID-19 potentially is a causal risk effect for AMI, and investigated the molecular mechanisms underlying their simultaneous occurrence using bioinformatics analysis. A brief research workflow is summarized in **Figure 10**.

### MR for genetic causal inference

The TSMR results indicated that neither COVID nor severe COVID-19 exhibits significant causal effects on the three AMI GWAS, suggesting that there is no evidence of genetic variation associated with COVID-19 influencing the occurrence of AMI, indicating the absence of a causal relationship. We applied the largest and latest available exposure and outcome GWAS statistics and rigorous screening criteria for IVs, but this MR study was based only on a European population and the IVs used for exposure may not be suitable proxies for inferring causality, although sensitivity analyses suggested that the results were generally reliable.

Furthermore, while MR is valuable for addressing confounding factors and unveiling causal relationships, it falls short of capturing the intricate biological processes that underlie disease interactions (24). In light of these considerations, we also conducted an investigation using public bioinformatics data to explore shared molecular biological mechanisms between COVID-19 and AMI. We identified two disease-shared genes, TLR4 and ABCA1, especially TR4, highlighting their potential central role involved in immune and inflammatory responses. We found that TLR4 and ABCA1 were associated with the infiltration of various immune cells and were highly expressed in CD16 monocytes in severe COVID-19 patients. These findings suggest that TLR4 and ABCA1 may play important roles in the development of COVID-19 with AMI.

### TLR4 in COVID-19 and AMI

Recent studies within the last decade have underscored the significance of immune and inflammatory responses in the advancement of atherosclerosis. This shift in understanding has transformed the characterization of acute coronary syndrome from lipid deposition to an inflammatory disorder (25–28). Viral infections have been shown to disrupt plaque stability, incite a cytokine storm, and prompt immune cell polarization toward an unstable phenotype (29, 30). Severe COVID-19 cases exhibit aberrant immune responses, compromised innate-adaptive crosstalk, and alterations in peripheral blood cell composition, including elevated CD14+ and CD16+ monocytes and reduced overall B cells (31, 32). The immune cell-expressed pattern recognition receptor (PRR) TLR4 plays a pivotal role in innate immunity and the inflammatory response to diverse pathogens (33, 34). It transcriptionally activates nuclear factor-κB (NF-κB) via myeloid differentiation primary response 88 (MyD88) or Toll/interleukin-1 (IL-1) receptor (TIR) domain-containing adapter-inducing IFN-β (TRIF), resulting in the generation of proinflammatory cytokines and chemokines, such as innate immune sensor genes, NOD-like receptor protein 3 (NLRP3) (35–37). Augmented TLR4 expression in COVID-19 has been linked to viral protein interactions, NF-κB activation, and cardiac complications like hypertrophy, inflammation, and fibrosis (38, 39). The manipulation of the TLR4 signaling pathway has emerged as a potential therapeutic avenue for mitigating COVID-19 complications (40, 41).

Distinctive features of severe COVID-19 cases in comparison to healthy controls and mild COVID-19 patients encompass heightened TLR4 activity (42). Our investigation likewise revealed an elevation in TLR4 levels in the blood samples of COVID-19 patients with AMI. The hyperinflammatory response instigated by TLR4 activation may provide a plausible rationale for AMI occurrences in COVID-19 patients.

TLR4, exclusively expressed on the cell membrane, plays a pivotal role in orchestrating the immune response against Gram-negative bacteria by recognizing bacterial lipopolysaccharides (LPS) (43). In the context of COVID-19, various TLRs can discern a wide array of infection-related elements, encompassing viral pathogen-associated molecular patterns (PAMPs) and host-derived damage-associated molecular patterns (DAMPs), to induce innate immune activation (37). Structural proteins and glycolipids from the SARS-CoV-2 virus are proposed as PAMPs capable of engaging TLR4, initiating an innate immune response, especially in the early stages of SARS-CoV-2 infection (43, 44). Recent findings have highlighted a direct interaction between the spike trimer of SARS-CoV-2 and TLR4, resulting in heightened TLR4 expression in monocytes (43), corroborating our scRNA-seq analysis. An in-silico suggests a stronger binding affinity of the SARS-CoV-2 spike protein to TLR4 when compared to other TLRs and the well-established entry receptor for SARS-CoV-2, angiotensin-converting enzyme 2 (ACE2) (11, 45). Additionally, *in vitro* assessments confirm that the spike protein trimer can induce the production of inflammatory cytokines through TLR4, akin to the effects of LPS stimulation (43). This trimer’s interaction with TLR4 may also upregulate ACE2 expression, potentially facilitating viral entry and exacerbating hyperinflammation (46). The interplay between the spike protein and TLR4, along with the heightened expression of genes linked to TLR4 signaling in COVID-19, underscores a captivating role for these receptors and their inflammatory cascade in disease etiology (23). However, the precise reasons behind TLR4’s robust recognition of SARS-CoV-2, which contains a substantial amount of single-stranded RNA (ssRNA), remain enigmatic. Theoretically, these ssRNA segments could be recognized by intracellular TLR7/8 to initiate antiviral immune responses (45, 47, 48). Nevertheless, the concept of immune responses primarily depending on host-virus interactions at the cell surface and downstream pro-inflammatory signal transduction suggests that intracellular TLR7/8 may not play a predominant role in this process (11, 45). The interactions between SARS-CoV-2 and TLRs are highly complex and warrant further investigation.

Positioned at the crossroads of thrombosis and the innate immune response, TLR4 emerges as a potential therapeutic target for SARS-CoV-2-related complications in light of the presented evidence (23, 46, 49).

### ABCA1 in COVID-19 and AMI

ABCA1, an ATP-binding cassette transporter highly prevalent in monocytes/macrophages, facilitates the elimination of cholesterol from foam cells, thereby exhibiting anti-atherosclerotic and cardioprotective effects (50, 51). Elevated ABCA1 levels have demonstrated potential cardioprotective attributes during AMI, while its deficiency may enhance immune cell activation and post-AMI repair processes (52). These observations highlight the multifaceted involvement of ABCA1 in AMI pathology.

Atherosclerosis, characterized by cholesterol accumulation in arterial walls leading to atherosclerotic plaques, constitutes a chronic inflammatory response. The process entails the continual infiltration of monocytes, which differentiate into macrophages and foam cells (28). Research indicates that the efflux of sterols facilitated by ABCA1 and ABCG1 influences the expression of inflammatory cytokines and chemokines in macrophages, as well as lymphocyte proliferative responses (53). Disruption of lipid transport due to the cytokine storm in COVID-19 might contribute to systemic inflammation. Modifications induced by COVID-19 in the quantity and composition of high-density lipoprotein (HDL) could diminish its anti-inflammatory and antioxidant characteristics, potentially leading to inflammation in virus-affected organs (54). Furthermore, genetic deficiency in ABCA1 has been linked to intensified inflammatory responses, particularly in the presence of LPS or other toll-like receptor (TLR) ligands that activate the TLR4/NF-κB pathway (55). Nonetheless, we found no significant increase in ABCA1 levels in PBMCs from clinical COVID-19 patients with AMI. Similarly, previous examinations probing ABCA1 expression in AMI patients have not unveiled substantial differences in ABCA1 mRNA and protein levels (56). Consequently, further experimental evidence is imperative to ascertain the potentially pivotal role of ABCA1 in COVID-19-related AMI.

### Strengths and limitations

This study has several strengths. First, it employs multiple methods and data sources to investigate potential causal links and molecular mechanisms between COVID-19 and AMI. Second, the findings provide novel insights into this topic from genetic and immunologic perspectives. Third, it provides potential biomarkers and therapeutic targets for the diagnosis and treatment of COVID-19-associated AMI. However, some limitations should also be recognized. Functional experiments were not conducted to verify the causal roles of TLR4 and ABCA1. The analysis was restricted to transcriptomic data from peripheral blood cells. Moreover, genetic variation and the impact of the molecular changes in AMI during COVID-19’s dynamic progression, including disease evolution and recovery, were not further explored. Furthermore, confining MR analysis to European populations restricts the generalizability of the conclusions worldwide, and it is essential to replicate the study using data from diverse racial and ethnic groups. Future GWAS studies employing diverse quantitative criteria could potentially establish whether COVID-19 is merely an incidental bystander or if it contributes to AMI development via intermediate factors. Investigating the functions of key molecules may enhance our comprehension of the immune dysregulation observed in COVID-19-related AMI, and thus provide prognostic indicators or therapeutic targets for clinical decision-makers.

## Conclusions

In summary, our MR analysis found no causal link between COVID-19 and AMI, indicating no statistical association between genetic variants influencing COVID-19 susceptibility and AMI development. Furthermore, we identify TLR4 and ABCA1 as potential contributors to the immune-related pathogenesis of COVID-19 with AMI. The dysregulation of TLR4 and ABCA1 may provide novel insights into the immune dysregulation and inflammatory response seen in COVID-19 and AMI cases.

## Data availability statement

The original contributions presented in the study are included in the article/**Supplementary Material**, further inquiries can be directed to the corresponding author/s.

## Ethics statement

The studies involving humans for the collection of clinical blood samples were approved by the ethics committee of the Lihuili Hospital Facilitated to Ningbo University (KY2022SL409-01). Informed consent was obtained from all recruited participants. The studies were conducted in accordance with the local legislation and institutional requirements. Written informed consent for participation in this study was provided by the participants’ legal guardians/next of kin.

## Author contributions

ZZ: Conceptualization, Data curation, Methodology, Resources, Software, Writing – original draft. YZ: Data curation, Investigation, Methodology, Resources, Supervision, Writing – original draft. YS: Data curation, Investigation, Methodology, Resources, Supervision, Writing – review & editing. PY: Methodology, Investigation, Validation, Writing – review & editing. XT: Conceptualization, Formal Analysis, Funding acquisition, Project administration, Supervision, Writing – review & editing.
