# Supplementary material for: Genetic and immunological insights into COVID-19 with acute myocardial infarction: integrated analysis of mendelian randomization, transcriptomics, and clinical samples
Source: Front Immunol. 2023 Nov 6;14:1286087. doi: 10.3389/fimmu.2023.1286087 (PMC10657900; doi:10.3389/fimmu.2023.1286087)
Supplement: Supplementary file 1 [file DataSheet_1.docx]

## Genetic and Immunological Insights into COVID-19 with Acute Myocardial Infarction: Integrated Analysis of Mendelian Randomization, Transcriptomics, and Clinical Samples

**SUPPLEMENTARY MATERIALS**


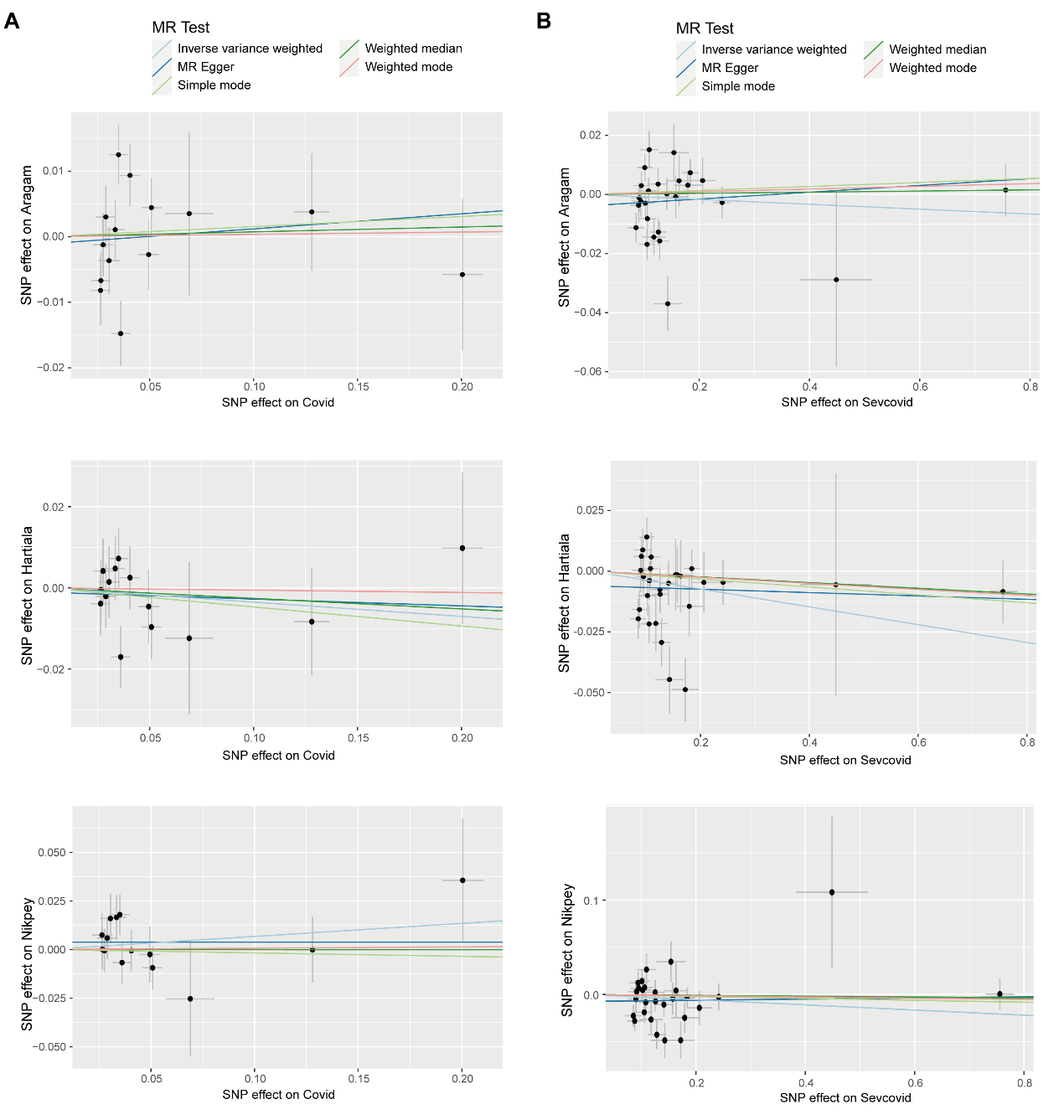


**Fig. S1** Mendelian randomization (MR) analysis of COVID-19 on AMI. (**A**) Scatterplot of individual variance regression coefficients for MR estimation of COVID-19 (Covid) on AMI. (**B**) Scatterplot of individual variance regression coefficients for MR estimation of severe COVID-19 (Sevcovid) on AMI.

**Tab. S1** Instrumental variables (IVs) with genome-wide significance in the COVID-19 (Covid) cohort.

| **Exposure** | | | | | | | | **Outcome** | | |
| --- | --- | --- | --- | --- | --- | --- | --- | --- | --- | --- |
| **Covid** | | | | | | | | **Aragam** | **Hartiala** | **Nikpey** |
| **SNP** | **EA** | **OA** | **beta** | **SE** | **F-statistics** | **P-value** | **EAF** | **P-value** | **P-value** | **P-value** |
| rs10774673 | T | C | 0.029 | 0.0048 | 35.840 | 2.14E-09 | 0.675 | 0.528 | 0.790 | 0.588 |
| rs1123573 | G | A | -0.026 | 0.0048 | 30.608 | 3.16E-08 | 0.379 | 0.116 | 0.619 | 0.500 |
| rs11264339 | T | C | -0.035 | 0.0046 | 58.943 | 1.62E-14 | 0.495 | 0.006 | 0.332 | 0.082 |
| rs12610495 | G | A | 0.050 | 0.0051 | 95.106 | 1.80E-22 | 0.303 | 0.615 | 0.615 | 0.860 |
| rs184781326 | G | A | -0.069 | 0.0117 | 34.925 | 3.42E-09 | 0.047 | 0.777 | 0.510 | 0.393 |
| rs2260685 | C | T | 0.033 | 0.0047 | 50.823 | 1.01E-12 | 0.474 | 0.815 | 0.546 | 0.152 |
| rs2290859 | T | C | -0.051 | 0.0049 | 109.404 | 1.32E-25 | 0.351 | 0.332 | 0.223 | 0.397 |
| rs2834158 | C | T | -0.041 | 0.0049 | 69.289 | 8.51E-17 | 0.661 | 0.048 | 0.748 | 0.955 |
| rs35044562 | G | A | 0.128 | 0.0085 | 227.972 | 1.64E-51 | 0.078 | 0.673 | 0.528 | 0.994 |
| rs676314 | G | A | 0.028 | 0.0048 | 32.808 | 1.02E-08 | 0.326 | 0.798 | 0.602 | 0.959 |
| rs679574 | G | C | -0.036 | 0.0046 | 61.526 | 4.37E-15 | 0.447 | 0.003 | 0.027 | 0.537 |
| rs7118388 | G | A | 0.027 | 0.0045 | 34.306 | 4.71E-09 | 0.506 | 0.129 | 0.947 | 0.995 |
| rs73062389 | A | G | 0.200 | 0.0099 | 413.143 | 7.61E-92 | 0.054 | 0.617 | 0.600 | 0.266 |
| rs9264740 | T | C | -0.030 | 0.0053 | 33.571 | 6.87E-09 | 0.732 | 0.476 | 0.870 | 0.211 |

**Tab. S2:** IVs with genome-wide significance in the severe COVID-19 (Sevcoivd) cohort.

| **Exposure** | | | | | | | | **Outcome** | | |
| --- | --- | --- | --- | --- | --- | --- | --- | --- | --- | --- |
| **Sevcovid** | | | | | | | | **Aragam** | **Hartiala** | **Nikpey** |
| **SNP** | **EA** | **OA** | **beta** | **SE** | **F-statistics** | **P-value** | **EAF** | **P-value** | **P-value** | **P-value** |
| rs10066378 | C | T | 0.118 | 0.0210 | 31.534 | 1.96E-08 | 0.116 | 0.017 | 0.059 | 0.097 |
| rs10850097 | T | C | 0.095 | 0.0149 | 40.775 | 1.71E-10 | 0.671 | 0.529 | 0.772 | 0.538 |
| rs11208559 | G | C | 0.103 | 0.0171 | 35.817 | 2.17E-09 | 0.291 | 0.542 | 0.228 | 0.677 |
| rs1123573 | G | A | -0.106 | 0.0152 | 48.825 | 2.80E-12 | 0.370 | 0.116 | 0.619 | 0.500 |
| rs1128175 | G | A | -0.126 | 0.0171 | 53.769 | 2.25E-13 | 0.753 | 0.518 | 0.409 | 0.859 |
| rs11614702 | A | G | 0.101 | 0.0138 | 53.704 | 2.33E-13 | 0.507 | 0.052 | 0.078 | 0.232 |
| rs117169628 | A | G | 0.157 | 0.0201 | 61.527 | 4.36E-15 | 0.137 | 0.915 | 0.896 | 0.797 |
| rs12534422 | T | C | 0.086 | 0.0151 | 32.269 | 1.34E-08 | 0.303 | 0.024 | 0.016 | 0.050 |
| rs12585036 | T | C | 0.141 | 0.0172 | 67.307 | 2.32E-16 | 0.213 | 0.968 | 0.589 | 0.403 |
| rs12610495 | G | A | 0.242 | 0.0160 | 226.751 | 3.05E-51 | 0.313 | 0.615 | 0.615 | 0.860 |
| rs12614007 | A | G | 0.094 | 0.0169 | 31.095 | 2.46E-08 | 0.749 | 0.663 | 0.320 | 0.317 |
| rs17279437 | A | G | -0.172 | 0.0251 | 46.945 | 7.29E-12 | 0.101 | - | 0.000 | 0.014 |
| rs17713054 | A | G | 0.756 | 0.0260 | 844.590 | 1.09E-185 | 0.075 | 0.865 | 0.518 | 0.979 |
| rs17885848 | T | C | 0.090 | 0.0156 | 33.331 | 7.78E-09 | 0.339 | 0.531 | 0.969 | 0.748 |
| rs2236645 | T | C | 0.179 | 0.0250 | 51.703 | 6.46E-13 | 0.085 | 0.524 | 0.243 | 0.124 |
| rs2569703 | G | C | -0.108 | 0.0140 | 59.400 | 1.28E-14 | 0.563 | 0.789 | 0.888 | 0.452 |
| rs28368148 | G | C | 0.449 | 0.0655 | 46.954 | 7.26E-12 | 0.023 | 0.326 | 0.903 | 0.177 |
| rs2897075 | T | C | 0.088 | 0.0144 | 37.465 | 9.32E-10 | 0.376 | - | 0.041 | 0.009 |
| rs343320 | A | G | 0.154 | 0.0275 | 31.435 | 2.06E-08 | 0.070 | 0.147 | 0.918 | 0.109 |
| rs34712979 | A | G | -0.110 | 0.0170 | 41.448 | 1.21E-10 | 0.254 | 0.015 | 0.564 | 0.122 |
| rs35705950 | T | G | -0.164 | 0.0228 | 51.555 | 6.95E-13 | 0.109 | 0.595 | 0.889 | 0.888 |
| rs368565 | T | C | 0.106 | 0.0149 | 50.168 | 1.41E-12 | 0.464 | 0.001 | 0.006 | 0.095 |
| rs41264915 | G | A | -0.206 | 0.0229 | 81.154 | 2.09E-19 | 0.094 | 0.544 | 0.707 | 0.437 |
| rs568035 | T | C | 0.143 | 0.0260 | 30.357 | 3.60E-08 | 0.063 | 0.000 | 0.001 | 0.009 |
| rs60132559 | T | C | 0.091 | 0.0150 | 37.130 | 1.11E-09 | 0.321 | 0.811 | 0.446 | 0.789 |
| rs61882275 | A | G | -0.126 | 0.0147 | 73.940 | 8.08E-18 | 0.343 | 0.005 | 0.211 | 0.492 |
| rs62056905 | G | A | -0.129 | 0.0168 | 58.358 | 2.18E-14 | 0.184 | 0.015 | 0.003 | 0.005 |
| rs9636867 | G | A | 0.184 | 0.0151 | 148.624 | 3.47E-34 | 0.331 | 0.105 | 0.892 | 0.747 |
