# Supplementary figures and images for: Genetic and immunological insights into COVID-19 with acute myocardial infarction: integrated analysis of mendelian randomization, transcriptomics, and clinical samples
Source: Front Immunol. 2023 Nov 6;14:1286087. doi: 10.3389/fimmu.2023.1286087 (PMC10657900; doi:10.3389/fimmu.2023.1286087)

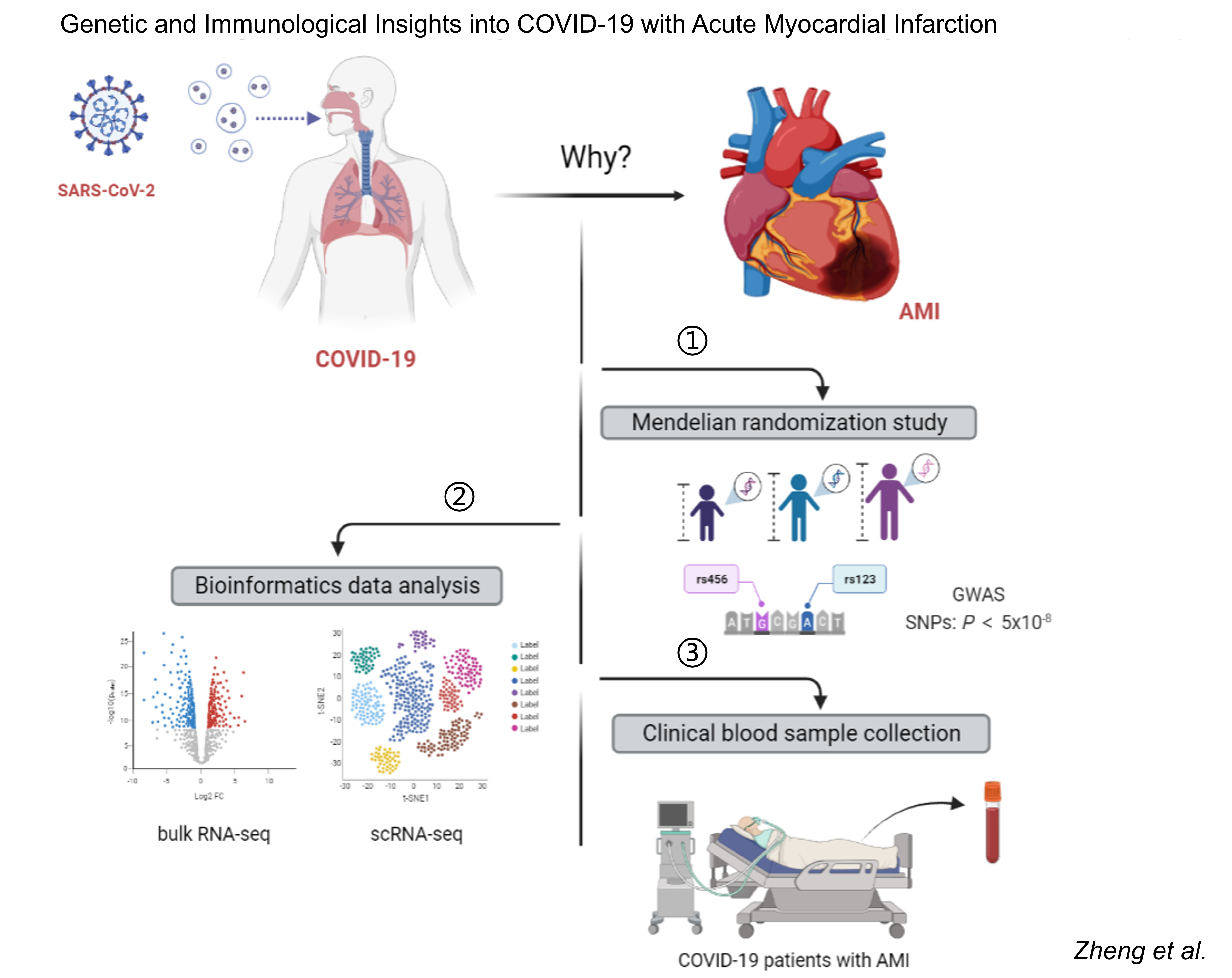

Supplement: Supplementary file 2 [file Image_1.tif]
